# Nosocomial outbreak of vancomycin-resistant *Enterococcus faecium* (VRE) ST796, Switzerland, 2017 to 2020

**DOI:** 10.2807/1560-7917.ES.2022.27.48.2200285

**Published:** 2022-12-01

**Authors:** Vanja Piezzi, Nasstasja Wassilew, Andrew Atkinson, Stéphanie D'Incau, Tanja Kaspar, Helena MB Seth-Smith, Carlo Casanova, Pascal Bittel, Philipp Jent, Rami Sommerstein, Niccolò Buetti, Jonas Marschall

**Affiliations:** 1 Department of Infectious Diseases, University Hospital Bern, University of Bern, Bern, Switzerland; 2 Department of Infectious Diseases, Lucerne Cantonal Hospital, Lucerne, Switzerland; 3 Division of Clinical Bacteriology and Mycology, University Hospital Basel, Basel, Switzerland and Applied Microbiology Research, Department of Biomedicine, University of Basel, Basel, Switzerland; 4 Institute of Medical Microbiology, University of Zurich, Zurich, Switzerland; 5 Institute for Infectious Diseases, University of Bern, Bern, Switzerland; 6 Department Health Sciences and Medicine, Clinic St. Anna, University of Lucerne, Lucerne, Switzerland; 7 Infection Control Programme, University of Geneva Hospitals and Faculty of Medicine, Geneva, Switzerland; 8 INSERM, IAME, Université Paris-Cité, Paris, France; 9 Division of Infectious Diseases, Department of Internal Medicine, Washington University School of Medicine, St. Louis, MO, United States

**Keywords:** Vancomycin resistant *Enterococcus faecium*, antibiotic resistance, VRE, nosocomial outbreak, outbreak management, infection prevention and control, Whole genome sequencing

## Abstract

A large clonal outbreak caused by vancomycin-resistant *Enterococcus faecium* (VRE) affected the Bern University Hospital group from the end of December 2017 until July 2020. We describe the characteristics of the outbreak and the bundle of infection prevention and control (IPC) measures implemented. The outbreak was first recognised when two concomitant cases of VRE bloodstream infection were identified on the oncology ward. During 32 months, 518 patients in the 1,300-bed hospital group were identified as *vanB* VRE carriers. Eighteen (3.5%) patients developed an invasive infection, of whom seven had bacteraemia. In 2018, a subset of 328 isolates were analysed by whole genome sequencing, 312 of which were identified as sequence type (ST) 796. The initial IPC measures were implemented with a focus on the affected wards. However, in June 2018, ST796 caused another increase in cases, and the management strategy was intensified and escalated to a hospital-wide level. The clinical impact of this large nosocomial VRE outbreak with the emergent clone ST796 was modest. A hospital-wide approach with a multimodal IPC bundle was successful against this highly transmissible strain.

**Key public health message**

**What did you want to address in this study?**

Vancomycin-resistant *Enterococcus faecium* (VRE) causes healthcare-associated outbreaks with severe infections in multimorbid or immunocompromised patients. We wanted to describe an outbreak caused by VRE ST796, with more than 510 affected patients during 32 months of outbreak activity in Switzerland, and understand its clinical impact. Then, we wanted to determine what countermeasures contributed to the control of the outbreak.

**What have we learnt from this study?**

Whole genome sequencing (WGS) was useful in identifying the sequence type of an isolate and determining the relationship between individual isolates. A multimodal hospital-wide infection control approach was successful in controlling the outbreak. This VRE outbreak was associated with a modest clinical impact.

**What are the implications of your findings for public health?**

In a non-endemic country, it is possible to control a large nosocomial *vanB* VRE ST796 outbreak. Because this emerging strain is highly transmissible, extended screening and countermeasures should be considered as soon as this VRE strain is identified in a patient.

## Background

Vancomycin-resistant *Enterococcus faecium* (VRE) emerged in the last decades as a multidrug-resistant microorganism with the ability to cause nosocomial outbreaks. This opportunistic pathogen typically only causes severe infections in multimorbid or immunocompromised patients. Even with adequate antibiotic treatment, invasive VRE infections are associated with increased mortality and longer hospital stays [1-3]. Transmission may occur via direct or indirect contact, the main reservoirs being asymptomatic carriers and contaminated surfaces [4].

In Europe, the rates of vancomycin resistance among invasive *E. faecium* isolates range from 0% to 50%, depending on the country [5]. Over the past decade, several small VRE outbreaks, mostly affecting tertiary care hospitals, have been documented in Switzerland [6-11]. According to the 2020 surveillance report by the Swiss Centre for Antibiotic Resistance (ANRESIS), Switzerland still has a comparatively low prevalence rate (1.7%) of vancomycin non-susceptibility in invasive *E. faecium* isolates [12]. However, a national investigation on enterococcal bacteraemias detected that the proportion of vancomycin resistance had increased from 0% in 2013 to 3.9% in 2018 among bacteraemic *E. faecium* [13].

## Outbreak detection

Starting with two cases of *vanB* VRE bloodstream infection diagnosed on the oncology ward on 30 December 2017, a large outbreak affected the Bern University Hospital Group for more than 2 years. The sequence type (ST) was ST796: this emergent clone had been described in 2012 in Australia and subsequently in New Zealand [14,15] and had not previously been encountered in Europe. Because of its rapid dissemination, ST796 is currently the third most frequent *E. faecium* clone causing VRE sepsis in Australia [16].

The purpose of this report is to describe the VRE ST796 outbreak in detail and to review the bundle of infection prevention and control (IPC) management strategies that were implemented, along with their practical limitations.

## Methods

### Study design and setting

We retrospectively describe a VRE outbreak that occurred between the end of December 2017 and July 2020 in a hospital group in Switzerland, a country otherwise non-endemic for VRE. This study follows the Outbreak Reports and Intervention Studies Of Nosocomial infection (ORION) guidelines for outbreak reporting [17].

The Bern University Hospital Group consists of one tertiary university hospital, four community hospitals and one rehabilitation centre, totalling 1,300 beds. More than 60,000 patients are admitted each year, resulting in almost 380,000 patient-days [18]. A dedicated IPC team operates for the entire hospital group.

### Population and statistical methods

We included all patients admitted to the hospital group between 30 December 2017 and 31 July 2020 who tested positive for *vanB* VRE (asymptomatic carrier or infected) and their contact patients. Table 1 describes the definitions used for outbreak characterisation. The relevant demographic information (age, sex), clinical (department, presence of infection) and microbiological details were prospectively extracted from the electronic medical records and from infection prevention databases.

**Table 1:** Definitions used for vancomycin-resistant *Enterococcus faecium* outbreak characterisation, Switzerland, 2017–2020

Term	Definition
*vanB* VRE	Vancomycin-resistant *Enterococcus faecium* (MIC > 4 mg/L), carrying the *vanB* gene
VRE-positive patient (PP)	*vanB* VRE carrier Patient with a positive culture (screening or clinical sample) for *E. faecium* resistant to vancomycin
	*vanB* VRE-infected Patient with a positive culture with diagnosis of an invasive infection and indication for antibiotic therapy
VRE contact patient (CP)	Patient hospitalised in the same room or on the same ward with *vanB* VRE-PP without adequate contact precautions (retrospectively identified going back 7 days of the stay of the index patient, in the early phase until October 2018 going back 30 days for the patients in the same room)
VRE transmission	New *vanB* VRE-PP from same ward as a known VRE-PP

CP: contact patient; MIC: minimum inhibitory concentration; PP: positive patient; VRE: vancomycin-resistant *Enterococcus faecium.*

Continuous variables are summarised as medians and interquartile ranges (IQR), categorical variables as percentages. All statistical analyses were performed using R (version 4.0.0).

### Microbiology

#### Culture- and PCR-based testing

Initially, the rectal swabs were inoculated in a selective enrichment broth (Enterococcosel Broth Becton Dickinson supplemented with 4.5 mg/L vancomycin, 2 mg/L meropenem, 16 mg/L amoxicillin) at 35 °C and incubated for up to 48 h. Subsequently, the broths were plated on selective and chromogenic plates (CHROMagarVRE, CHROMagar, France). We identified the species by matrix-assisted laser desorption/ionisation time-of-flight mass spectrometry (MALDI-TOF, Bruker-Daltonics, Germany) and confirmed the presence of *vanB* with the Xpert *vanA/vanB* assay (Cepheid, United States (US)). Phenotypic vancomycin resistance was assessed by Clinical Laboratory Standards Institute (CLSI) agar dilution [19] and by minimum-inhibitory-concentration testing (Etest, bioMérieux, France).

In July 2018, we introduced direct analysis by Xpert-*vanA/vanB* after swab inoculation in the selective enrichment broth for 20–25 h [20]. Thanks to the high negative predictive value of this diagnostic pathway, a subsequent culture according to the above procedure was performed only for unclear Xpert-*vanA/vanB* results or to confirm positive Xpert-*vanA/vanB* results. From October 2018, the commercial test was substituted with an in-house quadruplex PCR detecting vanA/B, an *E. faecium*-specific marker and an internal control [21-23].

#### Molecular typing

To describe the clonal relationship for the samples, we performed whole genome sequencing (WGS). As previously described [24], WGS was performed using a MiSeq Illumina platform (accredited with ISO 17025 norm at the Division of Clinical Microbiology, University Hospital Basel) with 2 × 300 nt paired-end sequencing after Nextera XT library preparation. After sequencing, the resulting reads were de novo assembled and analysed by core genome multilocus sequence typing (cgMLST) using Ridom SeqSphere Software (version 4.1.6). With the same software, the clonal relationship was illustrated with a minimum spanning tree. All read data have been deposited with the European Nucleotide Archive (ENA) under the project number PRJEB27159. 

## Results

### Outbreak epidemiology

Until 31 July 2020, all six sites constituting the Bern University Hospital Group in Bern, Switzerland, were affected by this VRE *vanB* outbreak. During 32 months, more than 27,000 screening samples were obtained (on average 200 samples per week) and 518 patients were found to be positive for *vanB* VRE (Figure).

**Figure:**
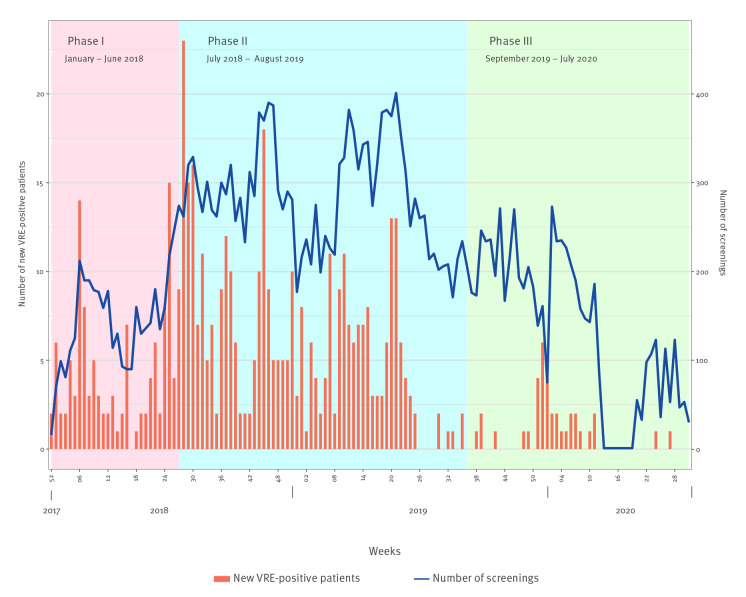
Epidemic curve of VRE detections (n =518) and screening volume (n = 27,725), Bern University Hospital Group, Bern, Switzerland, December 2017–July 2020

For a description of the three phases please see the chapter on Outbreak control measures.

Epidemiological characteristics of the VRE-PP are summarised in Table 2. Colonisation affected more men (n = 321; 62.0%) and occurred at a median age of 71 years (IQR: 61–80). Eighteen (3.5%) patients developed an invasive infection, of whom seven had a bloodstream infection and six an abdominal infection. Most infections (94.4%) occurred in the first 10 months of the outbreak. No deaths related to VRE infection were reported (i.e. 0% mortality rate).

**Table 2:** Epidemiological and clinical characteristics of patients colonised with *vanB* VRE, Bern, Switzerland, December 2017–July 2020 (n = 518)

	n	%
Median age in years (IQR)	71 (61,80)	
Male sex	321	62.0
Female sex	197	38.0
Type of screenings		
VRE-CP screening	415	80.1
Weekly ward screening	74	14.3
Admission screening	6	1.2
Hospital-wide screening	8	1.5
Clinical samples	14	2.7
Other	1	0.2
Invasive infection		
Bloodstream infection	7	1.4
Abdominal infection	6	1.2
Other invasive infection	5	1.0
No infection	500	96.5
Department		
Intensive care unit	42	8.1
Haematology-oncology wards	86	16.6
Abdominal surgery wards	46	8.9
Cardiovascular surgery wards	52	10.0
Other	292	56.4
Hospital type		
University hospital	373	72.0
Community hospital	127	24.5
Rehabilitation institution	18	3.5

CP: contact patient; IQR: interquartile range; VRE: vancomycin-resistant *Enterococcus faecium.*

The majority of positive samples (n = 373; 72.0%) were from the university hospital, where ca 940 of 1,300 beds in the hospital group are located. Forty-two (8.1%) of the patients were in the intensive care unit when they were screened, 8.9% (n = 46) on abdominal surgery wards and 16.6% (n = 86) in haematology-oncology.

A total of 415 (80.1%) of the VRE-PP were previously VRE-CP (i.e. they were either in the same room or on the same floor as a newly detected VRE case and thus epidemiologically linked), while another 74 (14.3%) were detected by weekly cross-sectional screening on high-risk wards and six (1.2%) through admission screening.

### Outbreak strain

The vanB VRE strain found during this outbreak shows an antimicrobial susceptibility profile with high susceptibility to teicoplanin (median minimum inhibitory concentration (MIC) of 0.5 μg/mL; IQR: 0.5–0.75 μg/mL; 307 isolates with teicoplanin MIC available for this analysis). The MIC for vancomycin varied between the isolates with a median of 24 μg/mL and an IQR of 12–48 μg/mL (307 isolates had information on vancomycin MIC).

On all samples throughout 2018 and for some samples from 2019, we performed WGS from a total of 328 (63.3%) patients (one isolate per patient). Of these, 313 isolates were clonal and separated by 0–3 alleles in the cgMLST analysis. These were all identified as MLST type ST796, with a single locus variation in one of these isolates. One patient carried a more diverse ST796 isolate, 52 alleles from the outbreak cluster. Two patients with ST796 carried other VRE, too (ST117 and a novel ST). The remaining 15 patients carried solely VRE of another ST (13 ST117, one ST17 and one ST555).

## Outbreak control measures

As a guidance for developing a management plan with countermeasures, we used a recent recommendation on how to control a VRE outbreak from the Netherlands [22]. 

### Outbreak management team and internal task force

The outbreak management team consisted of two IPC physicians and three specialised infection prevention nurses. The team was responsible for outbreak monitoring, management and internal communication.

The internal task force consisted of the director of the infection prevention programme, the chief medical officer, the chief nursing officer, the director of technology, the director of support services and a representative of the microbiology laboratory, in addition to the head of marketing and public relations. Their function was to coordinate the multiple activities to control the outbreak, approve the overall strategy and generate a communication plan for inside and outside our hospital group.

### Ward management

Once the VRE outbreak was declared, a temporary admission stop was implemented for the affected wards. Each ward with VRE patients was split into three zones: a VRE cohorting zone with VRE-positive patients (VRE-PP), an intermediate zone with VRE contact patients (VRE-CP) and a zone with newly admitted patients without previous VRE exposure. Staff was cohorted according to these zones wherever possible.

All VRE-PP and VRE-CP were placed under contact isolation precautions, ideally in a single room with dedicated bathroom. Cohorting in the same room was allowed for VRE-CP and for VRE-PP, separately. From June 2018, VRE-CP were managed without pre-emptive isolation precautions because the capacity for providing dedicated rooms had been exhausted.

### Screening policy

VRE screening was performed by obtaining rectal swabs. An internal analysis revealed that screening additional body sites (e.g. urines, wounds, abdominal drainages) did not increase detection of VRE in a given patient (Nasstasja Wassilew, personal communication, July 2018).

Weekly ward screenings were implemented once one or more transmissions were documented (and continued until no new VRE-PP were detected for 3 consecutive weeks) or when three or more VRE-PP were admitted concurrently. We also started with focused regular screenings on high-risk wards (intensive care and oncology) and have continued these until present.

After a potential exposure to VRE, at least three negative rectal swabs at weekly intervals were required to declare a VRE-CP as non-carrier. From June 2019, we accepted a single negative rectal swab to declare someone a VRE non-carrier, as long as the initial exposure had been > 6 months ago. A VRE-PP was no longer considered an active carrier when the follow-up swab 6 months after the last detection was negative. Subsequently, we ordered two additional weekly swabs in these patients so as not to miss false negatives.

Hospital-wide screenings of all patients hospitalised for at least 4 days in one of our hospital group’s sites were performed in October 2018, February 2019 and September 2019. Starting in October 2018, VRE was added to the standard admission screening procedures for patients returning after hospitalisation abroad or in south-western Switzerland (as these regions are known for increased prevalence of other multi-resistant bacteria such as meticillin-resistant *Staphylococcus aureus* and extended spectrum beta-lactamase-producing *Enterobacteriaceae*). From May 2019, we also started screening of patients transferred to our hospital group from other healthcare institutions in the Canton of Bern.

### Environmental screening

To test for environmental contamination, 30 samples were taken in August 2018 from patient rooms, medical equipment (e.g. ultrasound devices, computer keyboards) and nurses’ stations (especially work surfaces) from three affected wards, and those swabs were processed by culture by the clinical microbiology laboratory.

### Cleaning policy

We asked the respective manufacturers of all disinfectant products used in our sites (handrubs, disinfectant wipes and the surface liquid disinfectant, a quaternary ammonium compound) to test the activity of the products against VRE. 

The standard cleaning policy for the rooms consisted of daily cleaning of floors with a detergent, and an additional disinfection step (with Incidin Pro (Ecolab Life Sciences, US) 0.5%: quaternary ammonium compounds, phenoxyethanol and alkylamin) was applied upon discharge. From July 2018, the surfaces of patient rooms on wards with documented VRE cases were disinfected daily. From July 2018 to July 2019, daily environmental disinfection was extended to all patient rooms in the bed tower of the university hospital. This disinfection targeted the surfaces in patient rooms, floors and bathrooms including washbasins and toilets (products: Incidin Pro and different wipes with quaternary ammonium compounds and propanol).

From October 2018, we implemented (initially exclusively in the university hospital, from January 2019 also in the largest community hospital) an additional, terminal room cleaning procedure with UV-C light once the VRE-PP was discharged. The UVDI-360 Room Sanitizer, a single tower with 360 degrees of UV radiation, was placed in the empty room to disinfect all relevant surfaces.

Additional handrub dispensers were installed in key locations in the hospital starting mid-2019.

### Informatics support, electronic labelling

In the electronic medical records (EMR) and other information technology (IT) applications with patient data, we introduced VRE labelling based on a risk code assigned by IPC (e.g. VRE-PP, VRE-CP). Previously, the EMR had not displayed information such as isolation requirements on its overview page. In addition, the infection preventionists were able to automatically extract which VRE-CP needed to be screened thanks to a newly developed algorithm.

### Internal information and education

At the beginning of the outbreak, daily meetings with the ward teams were scheduled to coordinate activities. When the number of affected wards increased, a weekly information session was organised in the university hospital. In addition, the main strategic changes were always communicated by email. Educational material for hospital staff and patients was created and published on the hospital’s intranet, and we developed the first institutional screensaver with IPC content that was displayed on more than 10,000 computers across all sites. The IPC team visited the affected departments regularly and offered training and process audits. In addition, the entire workforce of more than 12,000 employees was requested to undergo an outbreak specific e-learning module. For patient instruction, we created a short videoclip about multidrug-resistant bacteria.

### Information outside the hospital

Family doctors were informed about their patient’s VRE status via the discharge report of the hospital. The receiving healthcare institutions were informed by telephone in addition to the written discharge report. We gave updates to and held regular meetings with the health authorities of the Canton of Bern and the National Centre for Infection Control (Swissnoso).

Table 3 summarises all the components of the outbreak management.

**Table 3:** Detailed overview of control measures implemented during the outbreak of vancomycin-resistant *Enterococcus faecium* ST796, Switzerland, 2017–2020

Timeline of interventions	Details of intervention
**Setting**	
Bern University Hospital Group, 1,300 beds: one tertiary referral centre, four community hospitals and one rehabilitation centre. Three ICU (two in the university and one in community hospital) with a total of 40 beds and a haematology–oncology ward with 45 beds. More than 60,000 admissions/year (2019), resulting in almost 380,000 patient-days per year (2019).	
**Diagnostics**	
December 2017–July 2018	Conventional culture
July–October 2018	Commercial vanB/vanA PCR with culture-confirmation (for unclear and positive samples)
	In September and October partially pooling PCR (four swabs for one PCR)
Since October 2018	In-house quadruplex PCR with culture confirmation
January–December 2018	WGS-based typing
**VRE isolation policy**	
December 2017–June 2018	Contact precautions for VRE-PP and VRE-CP. Cohorting of two or more VRE patients in the same room if identical resistance trait (*vanA* or *vanB*) and temporally close VRE identifications (< 6 months)
Since June 2018	Contact precautions only for VRE-PP
**VRE screening policy**	
***VRE-CP screening***	
December 2017–June 2019	Three screenings indicated with at least 7-day interval between them
Since June 2019	Only one new screening indicated if last exposure > 6 months ago
***Weekly ward screening***	
Since January 2018	ICU of university hospital and oncology wards until further notice
January 2018–July 2019	IMC of university hospital
June 2018–March 2020	ICU of largest community hospital
Since January 2018	Wards with documented transmission (stop of screening after 3 consecutive weeks without new VRE detections)
Since October 2018	Wards with > 2 VRE-PP (stop after 3 consecutive weeks without new VRE detection, from May 2019 switched to 1 week without new VRE detection)
***Admissions screening***	
January–May 2018	Oncology wards
January 2018	Two wards in internal medicine
Since October 2018	Admission screening for patients transferred from foreign countries and from south-western Switzerland
Since May 2019	Admission screening for patients transferred from healthcare institutions in the Canton of Bern
***Hospital-wide screening***	
October 2018–January 2019	First hospital-wide-screening
February 2019–May 2019	Second hospital-wide-screening
September 2019–November 2019	Third hospital-wide-screening
**Deisolation policy for VRE-PP**	
Since June 2019	One screening upon admission, if in the 6 previous months no detection and no hospitalisation. If negative, then stop contact precautions but maintain regular screening (1×/week during hospitalisation)
**Cleaning policy**	
January–July 2018	Intensified cleaning policy
July 2018–July 2019	Daily disinfection of the rooms in the bed tower of the university hospital and on the affected wards from the community hospital
Since October 2018	Additional UV-C decontamination in university hospital
Since January 2019	Additional UV-C decontamination in the largest community hospital
Since July 2019	Daily disinfection of the rooms only on the affected wards of the hospital group
**Additional infection control strategies during the study**	
***2018***	
January	Formation of outbreak management team and of the internal task force
	Starting the line list
	Additional handrub dispensers distributed
	First open information session
	Temporary admission stops on affected wards (stopped in June 2018)
February	First broad email information to all the clinical departments
June	First ward audit (overall, 10 departments were audited in the following months)
July	Introduction of ‘ward management’ guidance for affected wards
	Electronic identification of VRE-CP
August	Electronic labelling of VRE-PP and VRE-CP.
	Automated information about VRE-PP in discharge report
	Environmental screening
	Additional handrub dispensers placed
	Educational material for hospital staff made available
September	Chlorhexidine bathing in the ICU (September 2018–May 2019)
	Educational material for patients made available
	National VRE task force Swissnoso issues guidance paper [25]
October	Automated information about VRE-CP in discharge report
	All computers across the hospital group with screensavers with IPC content
***2019***	
February	Start Easy-Learn (e-learning module)
May	Educational videoclip for patients

CP: contact patients; ICU: intensive care unit; IMC: intermediate care; IPC: infection prevention and control; PP: positive patients; UV-C: ultraviolet C; VRE: vancomycin-resistant *Enterococcus faecium*; WGS: whole-genome sequencing.

### Phase I (January 2018 to June 2018): focalised outbreak

After the outbreak was declared on 4 January 2018, a VRE outbreak team was formed and a large outbreak investigation started. Focusing on the predominantly affected wards (oncology, internal medicine), a number of infection control measures were implemented immediately: contact precautions for VRE-PP und VRE-CP, admission screenings for all patients on affected wards, weekly ward screenings and temporary admission stops. As a result of these interventions, an initial decline of newly detected VRE-PP was observed.

### Phase II (July 2018 to August 2019): hospital-wide outbreak

Nonetheless, the outbreak flared up again in June 2018 with the same ST796 clone, as documented by cgMLST, now also involving previously unaffected departments. The management strategy was then escalated to hospital level. By means of a ‘heat map’ displaying the number of new VRE-PP in each department over time, the hospital leadership and the heads of the departments were regularly informed to raise awareness and foster accountability. Daily environmental disinfectant cleaning was implemented and intensified where VRE transmissions had been identified. Of the 30 environmental screenings performed in August 2018, only one was VRE-positive. Because of the lack of single rooms, we stopped the pre-emptive contact precautions for VRE-CP.

Because the need for screening increased substantially, the PCR Xpert *vanA/vanB* with a previous incubation step in an enrichment broth was introduced in summer 2018, a method that shortened the time to obtaining a negative result considerably. This change in diagnostics also enabled hospital-wide screenings. These point prevalence screenings, however, revealed a low yield (four positive samples with VRE *vanB* among 542 screenings and four among 609 in the first and second point prevalence screening, respectively) and confirmed that the outbreak was not occurring homogeneously across the hospital group.

With these measures, we observed a decrease in cases and clusters over the summer 2019.

### Phase III (September 2019 to July 2020): Control of the outbreak

In November 2019, we performed the third hospital-wide screening, with no *vanB* VRE discovered among the 621 individual screenings. Following a small cluster of *vanB* VRE in December 2019, new VRE detections occurred only occasionally, and no further transmission of the epidemic clone was observed after that.

We discontinued some implemented measures in a stepwise fashion: the daily room disinfection became again restricted to isolation rooms, and the frequency of informational meetings was reduced. In July 2020, in view of the period of sustained control we declared the outbreak to be over. The end of the outbreak was based on the criteria laid out in national guidelines [25]: no new case shall be detected for a period of 3 weeks after the identification of the last confirmed case, and there shall be an additional three negative department-wide point prevalence studies.

The regular screenings in the ICU and on the oncology wards continue as a risk reduction measure until further notice.

## Discussion

In this article, we describe the successful control of an outbreak caused by *vanB* VRE ST796 with more than 510 patients detected during 32 months of outbreak activity. Our most important findings were: (i) WGS is highly useful for identifying the ST of an isolate and determining the relationship between individual isolates; (ii) the initial IPC strategy that focused solely on affected wards was not sufficient for controlling the outbreak, but (iii) an expanded, hospital-wide outbreak management proved to be successful, and lastly (iv), when considering the infection rate of only 3.5%, the outbreak was associated with a modest clinical impact. Importantly, the workload of the IPC connected to this outbreak which involved large numbers of VRE-positive patients, did not allow us to identify each potential transmission event.

The ST796 strain, characterised by rapid and efficient spread, had previously been described exclusively in Australasia [14,15,26] (for a detailed complete genome description see [15]). To our knowledge, this was also the first documented outbreak with this ST in Europe. In recent publications from the neighbouring countries Germany [27-29] and Italy [30], molecular characterisation of local vancomycin-resistant *E. faecium* isolates failed to detect this new clone.

In the outbreak described here, WGS proved to be useful to describe the ST, to document the clonality, and to define the relationship between individual isolates and thus confirm transmissions. The crucial role of WGS in infectious disease surveillance was highlighted in a recent review on the detection and differentiation of *Enterococcus* spp. as was the subsequent identification of new strains and resistance genes [31]. However, owing to the associated costs and the considerable test turn-around time, we would suggest a targeted use of the WGS technology for large outbreaks. The sequencing of isolates is of interest especially in the early phase of detecting and investigating an outbreak, as well as for the evaluation of cases with no obvious epidemiological link. A Switzerland-wide molecular epidemiological surveillance platform is planned and may make WGS more accessible and affordable for non-academic institutions [32].

The initial IPC strategy of focusing on affected wards appeared to be insufficient in our setting (or at least did not result in a drop of cases as quickly as expected). Given that VRE spreads mostly in a silent fashion (that is, via carriers without symptoms of an infection), and VRE ST976 does this particularly rapidly when compared with other ST [14], the beginning of such an outbreak can easily be missed and its extent can be difficult to estimate. In the second phase of the outbreak, we expanded IPC measures to a hospital-wide level regardless of the known presence or absence of VRE, while continuing ward-specific measures. In the past, other outbreak reports have demonstrated the efficacy of expansive screenings similar to our strategy to detect VRE carriers [22,33]. This has been part of so-called ‘search and destroy’ strategies, where the detection of yet unknown pockets of transmissions is considered key in the control of an outbreak. In our setting, the low yield of the hospital-wide screenings was probably due to the rather late deployment in the course of the outbreak which in turn was the result of logistical difficulties. In our opinion, this approach is also helpful to confirm that the transmissions have abated and the epidemic wave has been broken, and to support the decision to scale back IPC measures.

Of note, this successful multimodal and hospital-wide strategy was obviously associated with time-consuming measures and substantial costs: we estimated that this outbreak generated costs of ca EUR 7 million, resources that are not necessarily available in all healthcare settings. As in other studies, the number of infections and related mortality was low [22,34,35]. Nevertheless, we postulate that the relevance of the implemented control measures was high, as we were able to demonstrate that they prevented the further spread of VRE in our low prevalence country [6,7]. We hypothesise that a much greater clinical burden of VRE disease could be avoided in this way. In addition, almost all the described infections caused by VRE occurred in the first third of the outbreak, suggesting that our intensified outbreak management with its apparent effect on the number of colonisations may have reduced the incidence of VRE infections as previously described by Ziakas et al. [36].

This outbreak description has several limitations. Firstly, as frequently seen in recommendations for outbreak control, the control measures detailed in this article were bundled, which makes it difficult to evaluate the impact of each individual containment measure on the control of the outbreak. Secondly, we ordered screenings of VRE-CP primarily if they were still hospitalised; after discharge, however, it was no longer possible to complete all the recommended screenings. Accordingly, because of incomplete follow-up of patients and under-reporting of cases, a selection bias is likely. Thirdly, VRE screening of employees was not a requirement, and very few employees asked occupational health for testing (data not shown). Therefore, we cannot make any statement on the role of healthcare workers in the transmission chain. Novel screening approaches based on machine learning were prototyped during the outbreak, and these are now in place to better determine who may have a link to other cases and should therefore be screened in similar nosocomial outbreaks in future [37,38]. Finally, given the costs for sequencing, we performed WGS only for approximately 1 year. Therefore, the molecular epidemiology data of the outbreak is incomplete. Still, the information based on 328 sequenced isolates is suitable for further analysis of VRE ST796 in terms of characterising factors that favour transmissibility and other molecular changes that may have occurred during the outbreak.

## Conclusion

We report the detection of a very large – and presumably the first – VRE ST796 outbreak in Europe. A hospital-wide and multimodal infection control bundle approach was successful in controlling the spread of this highly transmissible VRE ST796 in a non-endemic country.
